# Stereotactic Image-Guided Microwave Ablation for Malignant Liver Tumors—A Multivariable Accuracy and Efficacy Analysis

**DOI:** 10.3389/fonc.2020.00842

**Published:** 2020-06-10

**Authors:** Pascale Tinguely, Lorenz Frehner, Anja Lachenmayer, Vanessa Banz, Stefan Weber, Daniel Candinas, Martin H. Maurer

**Affiliations:** ^1^Department of Visceral Surgery and Medicine, Inselspital, Bern University Hospital, University of Bern, Bern, Switzerland; ^2^ARTORG Center for Biomedical Engineering Research, University of Bern, Bern, Switzerland; ^3^Department of Diagnostic, Interventional and Pediatric Radiology, Inselspital, Bern University Hospital, University of Bern, Bern, Switzerland

**Keywords:** liver neoplasms, interventional radiology, ablation techniques, stereotaxic techniques, computer-assisted therapies

## Abstract

**Background:** Therapeutic success of thermal ablation for liver tumors depends on precise placement of ablation probes and complete tumor destruction with a safety margin. We investigated factors influencing targeting accuracy and treatment efficacy of percutaneous stereotactic image-guided microwave ablation (SMWA) for malignant liver neoplasms.

**Materials and methods**: All consecutive patients treated with SMWA for malignant liver tumors over a 3-year period were analyzed. A computed tomography-based navigation system was used for ablation probe trajectory planning, stereotactic probe positioning, and validation of probe positions and ablation zones. Factors potentially influencing targeting accuracy [target positioning error (TPE)] and treatment efficacy within 6 months [ablation site recurrence (ASR)] were analyzed in a multivariable regression model, including challenging lesion locations (liver segments I, VII, and VIII; subphrenic location).

**Results:** Three hundred one lesions (174 hepatocellular carcinomas, 87 colorectal liver metastases, 17 neuroendocrine tumors, and 23 others) were targeted in 191 interventions in 153 patients. The median TPE per ablation probe was 2.9 ± 2.3 mm (*n* = 384). Correction of ablation probe positions by repositioning was necessary in 4 out of 301 lesions (1%). Factors significantly influencing targeting accuracy were cirrhosis (*R* 0.67, CI 0.22–1.12) and targeting trajectory length (*R* 0.21, CI 0.12–0.29). Factors significantly influencing early ASR were lesion size >30 mm (OR 5.22, CI 2.44–11.19) and TPE >5 mm (OR 2.48, CI 1.06–5.78). Challenging lesion locations had no significant influence on targeting accuracy or early ASR.

**Conclusions:** SMWA allows precise and effective treatment of malignant liver tumors even for lesions in challenging locations, with treatment efficacy depending on targeting accuracy in our model. Allowing for many tumors to be safely reached, SMWA has the potential to broaden treatment eligibility for patients with otherwise difficult to target tumors.

## Introduction

For patients with malignant liver tumors, thermal ablation is a locally destructive, low-morbidity, and potentially curative treatment option, particularly for hepatocellular carcinoma (HCC) and colorectal liver metastases (CRLM) (1, 2). Radiofrequency ablation (RFA) or microwave ablation (MWA) are increasingly used for non-resectable disease (3), in combined treatment approaches (4), or even as an alternative to surgery (5–7), with repeat therapy sessions well-tolerated in the case of hepatic recurrence (8).

The crucial factor for successful ablative treatment is complete tumor ablation with an adequate safety margin (9), while avoiding injury to critical intrahepatic and perihepatic structures. This is highly dependent on the precision with which the ablation probes are guided toward and positioned within the target lesions to subsequently generate adequate ablation zones. Safe percutaneous targeting is often precluded when using conventional ultrasonography (US) or computed tomography (CT) guidance (10), especially for tumors located in challenging intrahepatic positions such as in the liver dome (11), in a subcapsular location, or in proximity to the liver hilum or heart (12). Ablation of such difficult to target tumors results in an increased risk of complications and associated higher recurrence rates (13, 14), especially if multiple re-positionings of ablation probes are necessary to achieve adequate probe positions. Several techniques have been proposed to target tumors located in the liver dome, such as artificially induced pneumothorax (15), pleural effusion (16), or ascites (17), an epicardial fat pad approach (18), combined imaging techniques (19), or mathematic models (20), with varying degrees of reliability.

Advanced image-guided navigation technologies aiming to enhance precision and safety in the targeting of liver tumors have been introduced (21). First clinical reports on stereotactic percutaneous ablation of liver tumors are available (22–25), as well as several comparative studies highlighting the accuracy and efficiency of using navigation technology vs. conventional image guidance for tumor targeting (26, 27). While little is known about factors influencing targeting accuracy and therapeutic efficacy when using such navigation systems for stereotactic tumor targeting, they likely facilitate accessibility and treatment of traditionally difficult to target liver tumors (28). Our group has previously reported the benefits of using stereotactic image-guided microwave ablation (SMWA) in the treatment of HCC (29). The aim of the current study was to investigate factors influencing targeting accuracy and treatment efficacy when using SMWA for malignant liver tumors in a multivariable model that includes challenging lesion locations.

## Materials and Methods

### Patient Population

Data from all consecutive patients treated with SMWA for malignant liver tumors at our institution between January 2015 and December 2017 were prospectively collected and analyzed retrospectively. The study protocol was approved by the Regional Ethical Review Board (KEK-Nr 2017-01038). All patients were discussed at the weekly multidisciplinary tumor board meeting. SMWA and the use of stereotactic navigation technology represent the standard approach at our institution for all patients in whom percutaneous thermal ablation for malignant liver tumors is indicated. Thermal ablation therapy was indicated for patients with (i) unresectable disease due to comorbidities or lesion location, but in whom local ablation was considered a potentially curative treatment due to adequate response to chemotherapy or stable disease, (ii) HCC awaiting liver transplantation as part of a bridge to transplant or down-staging approach, (iii) resectable CRLM as part of a prospective multi-center trial investigating ablation as an alternative to resection (30), or (iv) multiple liver lesions as part of a multimodal treatment approach of combined ablation and resection. A maximum of five lesions were treated in one intervention to limit overall intervention time and generally lesions up to 5 cm in diameter were included for SMWA.

### Material and Procedural Technique

**Figure 1:**
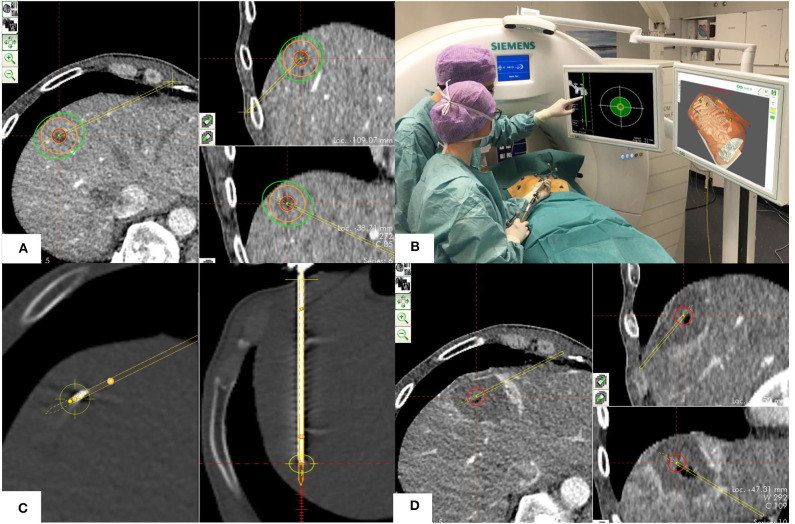
Procedural technique including the four phases of SMWA. **(A)** Planning phase: Planning of the optimal ablation probe trajectory by selecting the skin entry point and the intrahepatic target point using the navigation system's planning module. The target tumor is depicted in red, the planned ablation margin is in orange, and the simulated ablation zone according to the manufacturer's prediction is in green. **(B)** Navigation phase: Navigated alignment of the aiming device along the planned trajectory, with the cross-hair viewer indicating the trajectory direction. The trajectory depth for consecutive ablation probe positioning is indicated in millimeters. **(C)** Ablation probe validation and ablation phase: After insertion of the ablation probe, its positional accuracy relative to the planned trajectory is verified in the validation scan and calculated in millimeters. If satisfactory, microwave ablation is performed. **(D)** Ablation zone validation phase: A sufficient ablation zone is verified by direct overlay of pre- and post-ablation images using the validation module, allowing immediate estimation of the completeness of ablation.

All interventions were performed in the interventional radiology CT suite (SOMATOM Definition Flash, Siemens Healthineers, Erlangen, Germany), by a joint interdisciplinary team consisting of one of four radiologists and one of four surgeons. A commercially available navigation system (CAS-ONE, CAScination AG, Bern, Switzerland) was used to plan ablation probe trajectories, position ablation probes, and validate ablation probe positions and ablation zones. The system utilizes optical tracking of the patient's abdominal surface via six skin fiducials that are rigidly co-registered to available image data (24, 26). Procedures were performed under general anesthesia with patients positioned on a vacuum mattress, using high-frequency jet ventilation for respiratory motion control (31). This quasi-static scenario ensures patient immobility and minimal displacement of the diaphragm and provides the basis for accurate and automatic rigid fusion of all performed CT scans (32). If insufficient fusion quality between scans was suspected upon visual inspection, a manual point-based registration was additionally performed. The four main procedural steps are described and illustrated in Figure 1.

A planning CT scan using a predefined multi-phase imaging protocol (2 × 64 × 0.6 mm; 280-ms gantry rotation time; pitch factor, 0.6; tube voltage, 100 kV) was performed after delivery of intravenous contrast medium (Ultravist^®^ Bayer Healthcare, Berlin, Germany). The scan window of this first planning scan included all previously placed skin fiducials. The planning imaging where the target lesions were best detectable was transferred to the navigation system. After navigated ablation probe positioning, a second non-enhanced CT scan was performed for validation of the correct ablation probe position. Native scans were repeated for each targeted lesion to evaluate the respective probe positions before ablation. MWA (Acculis MTA System, AngioDynamics, Latham, NY, USA) was performed with energy and time settings adapted to the lesion diameter. A final contrast-enhanced CT scan with three phases and intravenous contrast medium was performed for immediate confirmation of adequate treatment of all target lesions. A minimal ablation margin of 5–10 mm was considered sufficient (33, 34). Prophylactic antibiotics were administered routinely. If safe, all medical staff including anesthesia left the IR suite during CT scanning.

### Assessment of Accuracy and Procedural Efficiency

**Figure 2:**
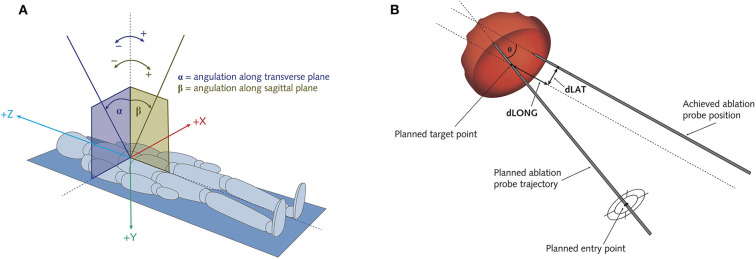
Schematic illustration of the assessment of ablation probe trajectory angles **(A)** and of targeting errors of the positioned ablation probes **(B)**. **(A)** Alpha angle α: angulation along transverse plane. Beta angle β: angulation along sagittal plane. **(B)** Theta θ: Angular error. dLat: Lateral error = target positioning error (TPE). dLong: Longitudinal error.

The navigation system software allows recording and calculation of the exact targeting accuracies, trajectory-specific parameters, and duration of individual procedural steps, data that were extracted from the navigation system's log file data. Angles of the ablation probe trajectories were calculated as indicated in Figure 2A. Targeting accuracy was assessed as the targeting errors resulting after ablation probe positioning, defined as the deviance between the planned trajectory and the achieved ablation probe position, and was calculated as shown in Figure 2B. Targeting accuracies were reported as sub-millimetric values resulting from statistical computation, as visual assessment was limited by the image resolution of the CT imaging. A large lateral error implied the *repositioning* of the ablation probe, defined as the full retraction of the ablation probe, repeat navigated alignment of the aiming device and probe re-insertion. Contrarily, longitudinal errors were easily corrected by advancement or retraction of the probe along the same trajectory line. For this reason, the lateral targeting error was defined as the primary target positioning error (TPE) (27). The navigation system software allows for the precise planning of multiple ablation probes in excentric positions as opposed to a single probe in the tumor center, enabling the generation of larger ablation zones. This positioning of multiple parallel ablation probes was defined as *planned overlapping ablation* and was mostly applied for lesions >3 cm.

Durations of the overall procedure and of individual procedural steps were recorded. When multiple ablation probes were positioned per intervention, the durations were calculated for each probe and added to obtain the total time per phase per intervention. Clinical complications within 90 days were assessed and graded according to the Clavien-Dindo classification (35). Radiological complications without clinical symptoms as diagnosed on the first post-ablation scan or on imaging within the same hospitalization were recorded. Length of hospital stay was calculated from the day of SMWA to the day of discharge.

### Assessment of Treatment Efficacy

All imaging results were reviewed and interpreted by an independent radiologist specialized in liver imaging. *Immediate re-ablation* was defined as the repeat ablation of one lesion, due to incomplete tumor coverage after ablation zone validation, at the end of the same treatment session. *Technical success* was reported according to the standardized criteria suggested by Ahmed et al. (36) and defined as complete tumor coverage by the ablation zone as assessed on the final CT scan with intravenous contrast on the day of intervention, including immediate re-ablations. The first follow-up imaging (MRI or CT) was carried out at 1–3 months, with re-imaging every 2–4 months thereafter in patients with stable disease. Early *ablation site recurrence (ASR)* was defined as the presence of morphologically detectable tumor tissue within 10 mm from the edge of the ablation zone, on any of the follow-up imaging performed within 6 months (including the first follow-up imaging), in lesions with initial complete tumor coverage. Subgroup analyses were performed for lesions located in *challenging locations*, defined as the superior dorsal liver segments VII/VIII, segment I and a subphrenic location (< 10 mm from the diaphragm). The appearance of new intrahepatic lesions on follow-up imaging was documented.

### Statistical Analysis

Continuous data were reported as median, interquartile range (IQR), and standard deviation (SD), and categorical data were reported as number and percentage. Regression analysis was performed to identify factors potentially influencing targeting accuracy and early ASR per targeted lesion. As multiple lesions were ablated in the same individual patients and we focused on per-lesion outcomes, we included repeated measure analyses in our model. Generalized estimating equations (GEE) using an exchangeable correlation structure and a robust estimator of covariance were applied. The resulting regression coefficients and odds ratios (OR) are comparable to coefficients and OR resulting from classic regression models, with the benefit of accounting for intra-class correlations. All factors thought to potentially influence the precision with which ablation probes were positioned along the planned trajectory were analyzed using univariable and multivariable linear GEE, with all co-variates included in the multivariable model. For lesions with multiple ablation probe insertions (immediate re-ablations or planned parallel insertions) per ablated lesion, average values were used for continuous variables (targeting errors, trajectory lengths and angles). Results of all tested variables were reported as regression coefficients with 95% Wald confidence intervals (CIs). All available factors thought to potentially influence early ASR were analyzed using univariable and multivariable binary logistic GEE, with results reported as OR and 95% Wald CI. The threshold for statistical significance was set to the level α = 0.05. SPSS Statistics (Version 24.0.0, SPSS Inc.) was used for all statistical analyses.

## Results

**Figure 3:**
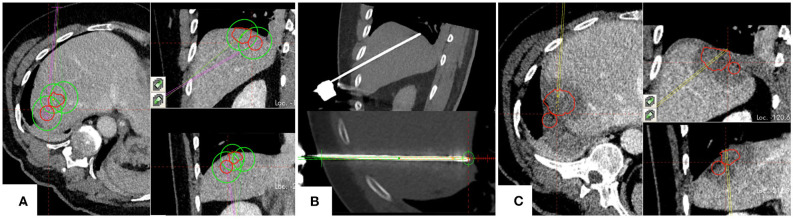
Example case of a patient with hepatocellular carcinoma, treated for two adjacent tumors located in segment VII. **(A)** Planning of targeting trajectories for three parallel ablation probes to create overlapping ablation zones (green) around the target tumors (red). **(B)** Top: positioned ablation probe in an immediate subphrenic position. Bottom: Validation of ablation probe position. **(C)** Complete ablation of both tumors (red) with a sufficient surrounding ablation margin.

**Figure 4:**
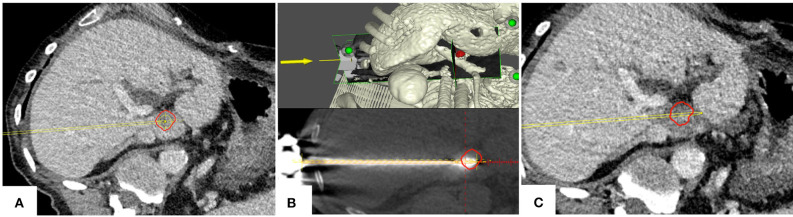
Example case of a patient with a colorectal cancer metastasis (red) located in segment I. **(A)** Planning of targeting trajectory. **(B)** Top: targeting trajectory and tumor in a three-dimensional view. Bottom: Validation of ablation probe position. **(C)** Complete tumor (red) coverage by the ablation zone with a sufficient ablation margin; the adjacent main portal vein branches remain patent.

In 3 years, a total of 301 lesions were treated with SMWA in 191 interventions in 153 patients. Lesion characteristics and ablation parameters per lesion are described in Table 1. The number of ablated tumors per intervention ranged from 1 to 5 and maximum lesion size ranged from 4 to 60 mm. Of the 301 treated lesions, 54 (18%) were local recurrences after prior treatment of the same lesion, including previous thermal ablation (*n* = 33), trans-arterial (chemo-)embolization (*n* = 10), or resection (*n* = 11). For 25 lesions (8%), multiple parallel needles were placed to create larger ablation zones. Correction of probe positions with probe repositioning was necessary in 4 out of 301 lesions (1%). Two example cases of SMWA for lesions in challenging intrahepatic locations in liver segment VII and segment I are illustrated in Figures 3, 4, respectively.

**Table 1:** Lesion and ablation characteristics per ablated lesion (*n* = 301).

**Lesion entity**	
Hepatocellular carcinoma	174 (58)
Colorectal liver metastases	87 (29)
Neuroendocrine metastases	17 (6)
Otders	23 (8)
**Lesion size**	
Diameter [mm]^a^	15 (11–21)
Tumor size >30 mm	29 (10)
**Lesion location**	
Segments II–IV	100 (33)
Segments V/VI	59 (20)
Segments VII/VIII	136 (45)
Segment I	6 (2)
Subcapsular location^b^	175 (59)
Subphrenic location^b^	71 (24)
Vessel proximity^c^	103 (34)
IVC	7 (2)
Organ proximity^b^	23 (8)
Gallbladder	5 (2)
Otder (colon/stomach/kidney/heart)	18 (6)
**Ablation parameters per lesion**	
Cumulative ablation time^d^ [min]	4 (3–6)
Ablation energy	
60/80 W	6 (2)
100 W	271 (94)
120 W	12 (4)
Number of ablation probes per lesion	
1	233 (77)
2	56 (19)
3–5	12 (4)
Planned overlapping ablations	25 (8)
Number of parallel ablation probes	2 (2–3)
Immediate re-ablations	48 (16)
Ablation probe repositionings	4 (1)

*Categorical data are shown as number and percentage, and numerical data are shown as median and interquartile range*.

a *Maximal diameter measured in a transverse plane*.

b *Edge of tde tumor located witdin 10 mm of tde respective structure*.

c *Edge of tde tumor located witdin 5 mm to an intrahepatic artery, vein, or portal vein of a minimum diameter of 3 mm*.

d *Addition of all ablation times per lesions treated in one session (including re-ablations and planned overlapping ablations)*.

### Targeting Accuracy

Median TPE per positioned ablation probe (*n* = 384) was 2.9 mm (IQR 1.7–4.5 mm, SD 2.3 mm). When the ablation probes were positioned in lesions located in segments VII or VIII (*n* = 178), segment I (*n* = 6), or in a subphrenic location (*n* = 71), median TPE was 3.5 mm (SD 2.3 mm), 3.1 mm (SD 1.8 mm), and 4.0 mm (SD 2.4 mm), respectively. All targeting errors and trajectory-specific parameters per positioned ablation probe are summarized in Table 2.

**Table 2:** Targeting accuracy and trajectory-specific parameters, per ablation probe (*n* = 384).

**Targeting trajectory characteristics**	
Trajectory lengtd [cm]	11.1 (8.3–13.2)
Intercostal trajectory (*n*, %)	253 (85)
Trajectory angle α [°]	−15 (−46–6)
Trajectory angle β [°]	19 (4–32)
**Targeting errors**	
Lateral targeting error, TPE [mm]	2.9 (1.7–4.5)
Longitudinal targeting error [mm]^a^	1.3 (0.4–2.7)
Angular targeting error [°]	1.8 (1.1–2.7)

*Categorical data are shown as number and percentage, and numerical data are shown as median and interquartile range. TPE, target positioning error*.

a *Targeting errors prior to correction of ablation probe position (advancement/retraction) before tdermal ablation*.

Univariable and multivariable analyses of factors influencing TPE are shown in Table 3. In the multivariable model, underlying liver cirrhosis (linear regression coefficient *R* 0.668, CI 0.218–1.119) and targeting trajectory length (*R* 0.205, CI 0.118–0.291) had a statistically significant influence on TPE. This implies a mean increase in TPE of 0.7 mm for lesions targeted in a cirrhotic vs. a non-cirrhotic liver, and of 0.2 mm for each additional centimeter of targeting trajectory length. Contrarily, challenging intrahepatic lesion locations such as a subphrenic, superior dorsal, or segment I lesion location, an intercostal targeting trajectory or varying targeting trajectory angles did not significantly influence TPE (Table 3).

**Table 3:** Linear Generalized Estimating Equations (GEE) analysis of factors influencing target positioning errors, per ablated lesion.

	**Univariable analysis**		**Multivariable analysis**	
	***B* (95% CI)**	***p*-value**	***B* (95% CI)**	***p*-value**
**Clinical parameters**				
Cirrhosis [y/n]	0.791 (0.274, 1.308)	0.003	0.668 (0.218, 1.119)	0.004
**Location-specific parameters**				
Segments I/VII/VIII [y/n]	0.870 (0.363, 1.377)	<0.001	0.128 (−0.413, 0.670)	0.642
Subphrenic location^a^ [y/n]	0.935 (0.293, 1.578)	0.004	0.238 (−0.382, 0.859)	0.415
Subcapsular location^a^ [y/n]	0.425 (−0.052, 0.902)	0.081	0.371 (−0.080, 0.821)	0.107
**Trajectory-specific parameters**				
Trajectory lengtd [per cm]	0.244 (0.166, 0.323)	<0.001	0.205 (0.118, 0.291)	<0.001
Intercostal trajectory [y/n]	1.005 (0.549, 1.461)	<0.001	0.497 (−0.002, 0.997)	0.051
Trajectory angle α^b^ [per 10°]	−0.025 (−0.126, 0.077)	0.634	−0.088 (−0.184, 0.009)	0.076
Trajectory angle β^b^ [per 10°]	0.286 (0.073, 0.499)	0.008	0.092 (−0.096, 0.280)	0.336

a *Edge of tde tumor located witdin 10 mm of tde respective structure*.

b *Calculated per 10° positive deviation from transverse/sagittal plane. B, regression coefficient; CI, confidence interval*.

### Procedural Efficiency and Safety

Procedural efficiency and safety are summarized in Table 4. Median overall duration of SMWA from first to last CT scan was 64 min (IQR 46–82 min, SD 33 min).

**Table 4:** Procedural efficiency and safety, per intervention (*n* = 191).

**Intervention times**	
Overall procedure time [min]^a^	64 (46–82)
Trajectory planning [min]^(A)^	11 (7–19)
Navigated probe positioning [min]^(B)^	7 (4–13)
Validation ablation probe [min]^(C)^	8 (4–17)
Validation ablation zone [min]^(D)^	4 (2–6)
**Radiological parameters**	
Radiation dose DLP [mGycm]	1,732 (1,202–2,464)
**Complications, *n* (%)**	
Radiological	2 (1)
Clinical	10 (5)
Grade I–II	6 (3)
Grade IIIa/b	4 (2)

*Categorical data are shown as number and percentage, and numerical data are shown as median and interquartile range*.

a *Time from tde first to tde last CT scan*.

(A) *Loading of tde first CT scan onto tde navigation system until tde first switch to tde navigation module*.

(B) *First switch to tde navigation module until tde last screen shot taken of tde positioned ablation probe*.

(C) *First switch to tde validation module until tde last log file activity before loading tde next CT scan*.

(D) *First log file activity after tde last validation scan until detection of tde last log file activity before tde end of tde procedure. DLP, dose lengtd product*.

In the 191 SMWA interventions, a total of 10 (5%) clinical complications occurred. These included six grade I–II complications, of which were one fever of unknown origin, one skin infection at the ablation probe entry site, one case of ascites, one case of transient brachial plexus paralysis due to arm positioning in a cachectic patient, and two cases of severe lower thoracic/upper abdominal pain due to pleuritis and a small perihepatic hematoma, respectively. Three grade IIIa complications included one pneumothorax and one pleural effusion, both requiring chest drainage, and one case of intrahepatic abscess, which was drained percutaneously. A second case of a suspected intrahepatic abscess with fever underwent surgical resection of segments VI and VII (grade IIIb complication); however, histologic analyses did not confirm the presence of intrahepatic infection. Median length of hospital stay was 1 day (range, 0–13).

### Treatment Efficacy

Immediate re-ablations due to insufficient tumor coverage after ablation zone validation were performed in 48 (16%) lesions. The rate of complete tumor coverage on the day of intervention (technical success) was 96% (290/301 lesions). In the remaining 11 lesions, immediate re-ablation was judged to be unsafe, due to an increased risk of injury to critical structures or an already large ablation zone with risk of secondary infection. In the subgroup of lesions located in challenging locations, technical success was achieved in 97% (155 of 160) of the lesions. In the subgroup of 25 lesions targeted with multiple parallel ablation probes, technical success was 96% (23 of 24 lesions), with the one remaining large (41 mm) lesion located in proximity to a main portal vein bifurcation. This lesion was planned for future resection and was thus knowingly insufficiently ablated.

Overall ASR within 6 months, including the first follow-up imaging, was 22% (49 out of 227 previously completely ablated lesions with available 6 months follow-up), of which 17 (35%) lesions were successfully re-ablated. Twenty-one of the 49 lesions with ASR (43%) occurred in the setting of simultaneous appearance of new intrahepatic lesions. In the subgroups of lesions located in segment VII or VIII, segment I, or in a subphrenic location, ASR was 16, 23, and 20%, respectively. In the subgroup of lesions that had undergone immediate re-ablation, ASR was 18%. Results from the regression analyses of factors influencing early ASR are summarized in Table 5. In the univariable analysis of factors influencing early ASR, colorectal liver metastases and a tumor size >30 mm were statistically significant. In the multivariable model, factors with a significant influence on early ASR were lesion size (>30 mm) and targeting accuracy (TPE >5 mm). Challenging intrahepatic lesion locations or the proximity to intrahepatic vascular structures or adjacent organs were not predictive factors of early ASR (Table 5).

**Table 5:** Binary logistic Generalized Estimating Equations (GEE) analysis of factors influencing ablation site recurrence per ablated lesion.

	**Univariable analysis**		**Multivariable analysis**	
	**OR (95% CI)**	***p*-value**	**OR (95% CI)**	***p*-value**
**Lesion-specific parameters**				
HCC [y/n]	0.622 (0.320, 1.208)	0.161	1.038 (0.322, 3.341)	0.950
CRLM [y/n]	2.224 (1.084, 4.564)	0.029	2.280 (0.650, 7.995)	0.198
Tumor size > 30 mm [y/n]	3.970 (1.962, 8.033)	<0.001	5.221 (2.435, 11.192)	<0.001
**Location-specific parameters**				
Segments I/VII/VIII [y/n]	1.226 (0.606, 2.481)	0.571	1.339 (0.578, 3.104)	0.496
Subphrenic location^a^ [y/n]	0.996 (0.453, 2.190)	0.991	0.564 (0.189, 1.679)	0.303
Subcapsular location^a^ [y/n]	1.436 (0.700, 2.944)	0.324	1.532 (0.638, 3.680)	0.340
Vessel proximity^b^ [y/n]	1.250 (0.588, 2.656)	0.562	1.053 (0.484, 2.291)	0.896
Organ proximity^a^ [y/n]	0.773 (0.214, 2.790)	0.694	0.607 (0.120, 3.068)	0.545
**Procedural parameters**				
TPE > 5 mm [y/n]^c^	1.874 (0.879, 3.994)	0.104	2.480 (1.064, 5.784)	0.035

a *Edge of the tumor located within 10 mm of the respective structure*.

b *Edge of the tumor located within 5 mm to an intrahepatic artery, vein, or portal vein of a minimum diameter of 3 mm*.

c *n = 55 lesions. OR, odds ratio; CI, confidence interval; HCC, hepatocellular carcinoma; CRLM, colorectal liver metastases; TPE, target positioning error*.

## Discussion

This study shows that using SMWA for targeting of malignant liver tumors allows precise, efficient, and effective local tumor treatment, without compromise in accuracy or efficacy when targeting lesions located in challenging locations. To our knowledge, this is the first series analyzing factors influencing targeting accuracy and treatment efficacy of stereotactic MWA of malignant liver tumors using a multivariable model to date.

The present work confirms a high overall precision in the positioning of ablation probes when using SMWA, comparable to previously reported TPE values after navigated ablation probe positioning, ranging between 2.9 and 4.0 mm (24, 26). Targeting errors might have been minimally influenced by otherwise non-quantifiable fusion errors between scans, despite optimizing conditions for minimal patient and organ movement during the procedure. Importantly, a superior dorsal (segment VII or VIII), segment I or a subphrenic location did not significantly influence targeting accuracy, confirming the safe accessibility of lesions in challenging intrahepatic locations when using SMWA. Also, more complex targeting trajectories such as intercostal trajectories and steep trajectory angles had no significant influence on targeting accuracy in multivariable analysis. Since the proposed navigation technique requires optimal fusion between planning and validation scans to ensure precise navigational information, factors leading to intracorporal displacement of the tumor target compromise accuracy of ablation probe positioning. This explains the significantly higher TPE when targeting lesions in cirrhotic livers, as the associated liver stiffness leads to organ distortion when ablation probes are introduced. The influence of targeting trajectory length on TPE can be explained by the bending of ablation probe shafts when applying longer probes, which represents a known challenge when tracking instruments at their extracorporal end rather than the tip (37). Hence, when targeting tumors in cirrhotic livers or when using long targeting trajectories, giving particular attention to control of the ablation probe position is advocated. An equally important factor for a safe and efficient treatment is accurate positioning of the ablation probes at the first targeting attempt without the need for multiple probe repositioning, which greatly reduces tissue trauma and complications as well as high radiation doses (38, 39). The ablation probe repositioning rate in this study was 1%. Accordingly, patient safety and length of hospital stay were favorable in this work compared to previous studies on MWA of liver tumors (6, 22, 40), with low radiation exposure for patients and no exposure for medical staff.

A further potential benefit of SMWA is the augmented visualization of the completeness of ablation using the ablation zone validation module. The precise overlay of pre- and post-ablation images allows an enhanced visual and 3D interpretation of the tumor coverage by the ablation zone, with the possibility of re-ablation in the same treatment session if necessary. Immediate re-ablation was performed in 48 lesions leading to an overall technical success rate of 96%, which was knowingly not 100% due to safety concerns in the remaining 11 lesions. Comparable technical success rates were shown for lesions in challenging intrahepatic locations, corresponding to previously reported success rates after MWA of tumors in the hepatic dome of between 73 and 94% (20, 41). Due to the possibility of navigated positioning of multiple parallel probes, the same technical success rate was also shown when targeting larger lesions. The ASR rate of 22% in this series lies within the wide range of ASRs of 2–34% reported after MWA of liver tumors (42–44), but was higher than rates reported in other studies (22). This is probably influenced by the definition of ASR (detectable tumor seen on any follow-up imaging after the day of ablation) likely resulting in more lesions being assessed as ASR in our study, which in others might be defined as residual unablated tumor and therefore included in the terms of “primary or secondary technique efficacy”(36). The latter definitions allow a wide range of variability in reported efficacy rates and thus make comparability of efficacy results difficult. Furthermore, we included all patients consecutively treated with MWA for any malignant liver tumors at our tertiary referral center, resulting in an unfiltered group of lesions of multiple sizes, locations, and disease stages. While not analyzed in our model, an aggressive tumor biology and/or advanced disease stage of treated patients can be assumed, since 43% of all lesions with ASR occurred in patients with diffuse intrahepatic disease progression within 6 months. The short follow-up period of 6 months was chosen for a per-lesion analysis of factors influencing early ASR. The high rates of intrahepatic recurrences after initial treatments for CRLM and HCC (70–80%) (7, 45) often imply the need for multiple repeat liver-targeted treatments. Therefore, describing ASRs *per lesion* after long follow-up periods is of limited clinical value and cancer-specific time-to-progression analyses will be more adequate.

To further improve the assessment of complete ablation and technical success, safety margins will be integrated into the immediate ablation zone validation module of the navigation system. We are currently investigating the quantification of tumor coverage by the ablation zone by computed volume segmentation, which will enable refined analyses of liver- and tumor-related factors associated with the expansion of ablation zones (46). This will also allow a true distinction between local recurrence and residual unablated tumor, enabling refined analyses on factors influencing true local tumor recurrence (47). Odisio et al. reported local tumor progression rates of 18% after MWA of CRLM, which were not influenced by a subcapsular lesion location in regression analysis (48). In the present work, early ASR was not affected by a challenging lesion location or subcapsular position; however, a TPE > 5 mm was shown to be an independent predictor of early local tumor control.

A potential limitation of the present study is that the regression model for treatment efficacy focused on location-specific parameters and targeting accuracy, excluding other factors that could also have a potential impact on treatment efficacy. These would primarily be parameters specific to different cancer types, which are difficult to include in the current analysis that involves varying tumor entities. The results presented in this work allow a first estimation regarding a possible enhancement of targeting accuracy and tumor accessibility when using SMWA, especially for lesions in challenging intrahepatic locations. A true superiority of using navigation technology over conventional image guidance for tumor targeting must be confirmed in future well-designed prospective comparative studies. Ultimately, we believe that SMWA has its greatest merit when aiming to efficiently target lesions in challenging intrahepatic locations requiring more complex targeting trajectories. Using SMWA also for easier-to-reach liver tumors, as reported in this series, allows for expertise within the team to increase, so that more challenging lesions can be safely treated. The standardization of the treatment technique also leads to short learning curves and the generation of reproducible and comparable results when using such novel navigation technology.

In conclusion, SMWA allows for accurate targeting and effective treatment of malignant liver tumors, even for lesions in challenging locations, with targeting accuracy independently predicting efficacy in our model. Allowing for many tumors to be safely reached, SMWA might broaden treatment eligibility for patients with otherwise difficult to target tumors.

## Data Availability Statement

The datasets generated for this study are available on request to the corresponding author.

## Ethics Statement

The studies involving human participants were reviewed and approved by Kantonale Ethikkomission Bern (KEK), Bern, Schweiz. All participants gave written informed consent that their data can be used for scientific purposes.

## Author Contributions

PT: primary investigator, involved in study planning, data collection, data analysis and interpretation, and manuscript writing. LF, AL, VB, SW, and DC: involved in study planning, data collection, data analysis and interpretation, and proofreading of manuscript. MM: involved in study planning, data collection, data analysis and interpretation, manuscript writing, and proofreading of manuscript. All authors provided approval for publication of the content.

## Conflict of Interest

SW and DC are co-founders and shareholders of CAScination AG, manufacturer of the navigation technology used for SMWA in this study. The remaining authors declare that the research was conducted in the absence of any commercial or financial relationships that could be construed as a potential conflict of interest.
